# Identification and expression analyses of *WRKY* genes reveal their involvement in growth and abiotic stress response in watermelon (*Citrullus lanatus*)

**DOI:** 10.1371/journal.pone.0191308

**Published:** 2018-01-16

**Authors:** Xiaozhen Yang, Hao Li, Yongchao Yang, Yongqi Wang, Yanling Mo, Ruimin Zhang, Yong Zhang, Jianxiang Ma, Chunhua Wei, Xian Zhang

**Affiliations:** 1 State Key Laboratory of Crop Stress Biology for Arid Areas, College of Horticulture, Northwest A&F University, Yangling, China; 2 Wenshan Academy of Agricultural Sciences, Wenshan, China; 3 Hanzhong City Agro-technology Extension Center, Hanzhong, China; 4 Yangtze Normal University, Fuling, China; Universidade de Lisboa Instituto Superior de Agronomia, PORTUGAL

## Abstract

Despite identification of *WRKY* family genes in numerous plant species, a little is known about *WRKY* genes in watermelon, one of the most economically important fruit crops around the world. Here, we identified a total of 63 putative *WRKY* genes in watermelon and classified them into three major groups (I-III) and five subgroups (IIa-IIe) in group II. The structure analysis indicated that *ClWRKY*s with different WRKY domains or motifs may play different roles by regulating respective target genes. The expressions of *ClWRKY*s in different tissues indicate that they are involved in various tissue growth and development. Furthermore, the diverse responses of *ClWRKY*s to drought, salt, or cold stress suggest that they positively or negatively affect plant tolerance to various abiotic stresses. In addition, the altered expression patterns of *ClWRKY*s in response to phytohormones such as, ABA, SA, MeJA, and ETH, imply the occurrence of complex cross-talks between *ClWRKY*s and plant hormone signals in regulating plant physiological and biological processes. Taken together, our findings provide valuable clues to further explore the function and regulatory mechanisms of *ClWRKY* genes in watermelon growth, development, and adaption to environmental stresses.

## Introduction

Transcription factors (TFs) play an essential role in regulating the transcription of specific target genes by binding to their promoters. The WRKY gene family is one of the 10 largest transcription factor families in plants [1]. Typically, proteins in this family possess one or two highly conserved WRKY domains which include a conserved WRKYGQK heptapeptide at N-terminus and a distinctive zinc-finger like motif C_2_H_2_ or C_2_HC at C- terminus [2, 3]. To regulate gene expression, the WRKY domain binds to the *cis*-acting element W box (TTGACC/T) in the promoter of the target gene [4]. In addition to W box, WRKY proteins can also bind to other elements, such as a sugar-responsive (SURE) *cis*-element (TAAAGATTACTAATAGGAA) and a pathogen-responsive element PRE4 (TGCGCTT), indicating the multiplicity in the mechanism of their functions [3].

Since the first cloning and characterization of the WRKY cDNA, *SPF1* from sweet potato [5], numerous *WRKY* gene families have been analyzed from more than 100 other plant species [6]. In *Arabidopsis*, 72 *WRKY* genes were classified into three groups I-III based on the number of WRKY domains and the features of their zinc-finger structure [2]. Group I proteins contain two WRKY domains, one at the C- and the other at the N-terminus, whereas group II and group III proteins only contain one single WRKY domain at N-terminus. Both group I and group II proteins have the same pattern of potential zinc ligands (C-X_4-5_-C-X_22-23_-H-X-H), while, Group III proteins contain one C_2_-H-C zinc-finger motif. Furthermore, group II proteins can be accurately divided into five subgroups (IIa+b, IIc, IId+e) based on the phylogenetic data of the WRKY domains [2, 7]. Phylogenetic analysis has proven that the groups II and III originated from the oldest group I proteins [7, 8], and the group II genes are not monophyletic [3]. In addition, genomic comparison from a model species to a less-studied species can provide important information on the expansion and the evolution of the *WRKY* gene families in plants [9].

Numerous studies have established the important roles of WRKY proteins in various physiological processes such as seed germination [10], lateral root formation [11], flowering time [12], fruit ripening [13], leaf senescence [14], and metabolic processes as well [15, 16]. WRKY proteins also play important regulatory roles in plant defense against various biotic and abiotic stresses, such as pathogens, nutrient deficiency, UV-B, heavy metals, salinity, drought and cold stress [17–19]. For instance, overexpression of *BnWRKY33* enhances resistance to *Sclerotinia sclerotiorum* in transgenic oilseed rape [20], while, double mutants of *AtWRKY54* and *AtWRKY70* in *Arabidopsis* clearly show enhanced tolerance to osmotic stress due to improved water retention and stomatal closure [21]. Notably, the regulatory roles of WRKY proteins are closely associated with multiple plant hormone-mediated signal pathways. In rice, various phytohormone treatments significantly alter expression patterns of 54 *WRKY* genes [22]. *AtWRKY50* and *AtWRKY51* work as positive regulators in the salicylic acid (SA) signaling pathway but as negative regulators in jasmonic acid (JA) signaling [23]. Increasing number of studies show that abscisic acid (ABA) signaling pathway is involved in the WRKY proteins-mediated responses of plants to various abiotic stresses [24–26].

The watermelon (*Citrullus lanatus* (thunb.) Matsum. & Nakai) is one of the most economically important fruit crops in the world. According to FAOSTAT2014, watermelon is cultivated all over the world with total area of 3.48 million hectares and annual production of 11.10 million tons, making it among the top five most consumed fresh fruits (http://www.fao.org/). Since a high-quality draft genome sequence of the East Asia watermelon cultivar 97103 has been reported [27], many transcription factors including mitogen-activated protein kinase (MAPK), no apical meristem-ATAF1/2-cup shaped cotyledon (NAC), and nuclear factor Y (NF-Y) have been subsequently identified and analyzed in watermelon [28–30]. However, there is still little information about *WRKY* genes in watermelon and their responses to environmental stresses and plant hormones. In this study, we performed a genome-wide identification of *ClWRKY*s in watermelon and analyzed their classification, chromosome distribution, phylogeny, structure, duplication, conserved motifs, and expression patterns in different tissues. Moreover, we further investigated the expression profiling of *ClWRKY* genes in response to abiotic stresses and plant hormones to exploit their potential functions in abiotic stress tolerances. Our study identified a subset of potential candidate *ClWRKY*s which can be utilized for enhancement of stress tolerance in *Cucurbitaceae* through genetic manipulation and rational breeding.

## Materials and methods

### Identification and annotation of *WRKY* genes in watermelon

To genome-wide identify *WRKY* genes in watermelon genome, both BLAST and Hidden Markov Model (HMM) methods were used in this study. Firstly, 22 and 102 WRKY proteins identified from *Arabidopsis* (https://www.arabidopsis.org/) and rice (http://rice.plantbiology.msu.edu/) were used as query sequences to search against the watermelon protein database (Version1, http://www.icugi.org) using BLASTP program with default settings. In addition, the HMM profile of the WRKY DNA-binding domain (Pfam: PF03106) downloaded from Pfam database (http://pfam.xfam.org/) was also exploited for identification of *WRKY* genes from watermelon using HMMER3.0 with E-value setting to 1e^-2^ [31]. Then, all putative non-redundant candidates were further subjected to identify partial or intact WRKY homologs in watermelon genome using TBLASTN methods (E-value setting to 1e^-10^). After parsing the BLAST files with in-house perl scripts, the new homologs were validated using non-redundant protein database from NCBI, and only sequences with best hit of WRKY protein were considered as candidate genes. Finally, all non-redundant putative *WRKY* genes were examined by the presence of WRKY domains using Conserved Domain Database (http://www.ncbi.nlm.nih.gov/cdd/), Pfam and ScanProsite (http://prosite.expasy.org/scanprosite/).

Using the software JoinMap 4.0 (https://www.kyazma.nl/index.php/mc.JoinMap/), the distribution of watermelon *WRKY* genes were constructed based on their chromosomal locations. Additionally, the molecular weight (MW), Theoretical isoelectric point (pI), instability index, aliphatic index, Grand average of hydropathicity (GRAVY) of watermelon WRKY proteins were predicted via the ProtParam tool from ExPASy (http://web.expasy.org/protparam/). An advanced protein subcellular localization prediction tool WoLF PSORT (https://wolfpsort.hgc.jp/) was used to predict the subcellular localization.

### Multiple sequence alignment, classification, and phylogenetic analysis

The protein sequences of *ClWRKY* genes obtained from watermelon, as well as 7 *AtWRKY* genes (*AtWRKY6*, *AtWRKY11*, *AtWRKY22*, *AtWRKY25*, *AtWRKY56*, *AtWRKY60* and *AtWRKY66*), were aligned using software MUSCLE [32], and visually edited by GENEDOC to analyze the conserved WRKY core domain (60 amino acid). A further multiple sequence alignment of 184 complete protein sequences (listed in S1 Table) including 57 *ClWRKY* genes from watermelon, 72 *AtWRKY* from *Arabidopsis* and 55 *CsWRKY* from cucumber, was performed using MUSCULE. Based on the alignment, a neighbor-joining phylogenetic tree was constructed using MEGA 7.0 with 1000 bootstrap value and Jones-Taylor-Thornton (+ G) method [33]. An online software iTOL was applied to beautify the phylogenetic tree (http://itol.embl.de/).

### Gene structure analysis and identification of motifs

The exon-intron organization of the watermelon *WRKY* genes were generated by comparing their coding sequences (CDS) with their respective full-length sequences (http://cucurbitgenomics.org/) using the online program Gene Structure Display Server (GSDS: http://gsds.cbi.pku.edu.cn) [34]. The conserved motifs of ClWRKY proteins were analyzed online using Multiple Expectation Maximization for Motif Elicitation (MEME) with default parameters (http://meme-suite.org/tools/meme).

### Gene duplication and synteny analysis

Duplication pattern and synteny analysis were performed following the procedures described previously [35]. All watermelon WRKY protein sequences were searched against themselves and proteins of *Arabidopsis* respectively, using BLASTp program with E-value setting to 1e^-10^ and output format as tabular (-m 8). Then, the destination tabular file, as well as the GFF files of watermelon and *Arabidopsis* genomes, were inputted into software MCScanX to analyze duplication types and syntenic relationship [36], and visualized using CIRCOS (http://circos.ca/).

### Plant material and treatments

The seeds of watermelon inbred line ‘Y34’, a typical East Asia ecotype were provided by the Cucurbits Germplasm Resource Research Group at Northwest A&F University, Yangling, Shaanxi, China. For tissue-specific analysis, germinated seeds were directly sown in the experimental base at Northwest A&F University, Yangling, Shaanxi, China (34°20′N, 108°24′E), and the roots, stems, leaves, tendrils, fruit, male and female flowers were sampled separately during the fruit maturation period. For other treatments, germinated seeds were sown in plastic pots (8 cm × 7 cm × 7 cm) filled with commercial peat-based compost (Shaanxi Yufeng Seed Industry Co., Ltd., Yangling, China). The seedlings were grown under spring time natural light in a greenhouse at Northwest A&F University, where the temperature was 28–35°C /16–20°C (day/night). All plants were uniformly watered daily and nourished weekly with 1/2 strength Hoagland’s solution. Seedlings at the four-week stage were used for the following treatments.

Hormone treatments were performed by spraying leaves with 100 μM ABA [28], 1 mM SA [37], 100 μM methyljasmonate (MeJA), and 10 mM ethephon (ETH) [38], while leaves sprayed with distilled water served as control. Leaves were collected at 0.5, 1, 6, 12, 24, and 48 hours after treatments.

The salinity stress treatment was carried out by irrigating plants with 300 mM NaCl solution (80 mL per plant) in the pots [39], followed by sampling leaves at 6, 24, 48, 72, 96, and 120 hours after treatment. Similarly, plants irrigated with distilled water were used as control. For cold treatment, plants were kept in a growth chamber at 4°C under a light intensity of 300 mmol•m^–2^•s^–1^ PPFD [40], then leaves were sampled at 1, 3, 6, 12, 24, and 48 hours after the commencement of cold treatment, whereas seedlings kept at 27±1°C under the same light condition were used as control. The drought stress treatment was accomplished creating a natural drought condition [41], and the control plants were well-watered to 70 ± 5% field capacity based on weighing. Then the drought-stressed and control leaves were sampled at 24, 48, 96, and 192 hours after treatment. In our study, at each time point of each treatment, the topmost second fully expanded leaves from four plants were pooled together in each biological sample, and three biological replicates were used in all treatments. Harvested samples were rapidly frozen in liquid nitrogen and stored at -80°C for further analysis.

### RNA isolation and semi-quantitative reverse-transcription PCR analysis

Total RNA of sampled leaves was extracted using the RNASimple Total RNA Kit (DP432, TIANGEN, China). The integrity and quality of RNA were analyzed via 1.0% agar gel electrophoresis and the NanoDrop 2000C Spectrophotometer (Thermo Fisher Scientific, USA). Approximately 1 μg of total RNA was used for the synthesis of first strand of cDNA by the FastKing RT Kit with gDNase (KR116, TIANGEN, China). Gene-specific primers for the *ClWRKY* genes were designed using Primer Premier 6.0 (S2 Table). Semi-quantitative reverse-transcription (RT) PCR (LifeECO-TC96, BIOER, China) was done following the PCR procedures: initial denaturation at 94°C for 90 s, followed by 40 cycles of denaturation at 94°C for 30 s, annealing at 58 ± 5°C for 30 s, extension at 72°C for 30 s, and a final extension at 72°C for 5 min. The amplification was done in a 20 μl reaction volume, which contained 10.0 μl 2×Taq Master Mix (E005, novoprotein, China), 0.6 μl of each primer (10 μM), 1.0 μl cDNA template, and 7.8 μl ddH_2_O. Meanwhile, a housekeeping gene *β-actin* gene (*Cla007792*) was used as internal control [42]. All PCR products were measured on a 2.0% agarose gels and imaged under UV light (ChampGel 6000, SAGECREATION, China) for gene expression analysis.

### Real-time quantitative PCR

The quantitative RT-PCR was conducted on a LightCycler^®^ 96 real time-PCR machine (Roche, Switzerland) using SYBR^®^ Premix Ex Taq^™^ II (TaKaRa Biotechnology, Dalian, China). The amplification was done in a 20 μl reaction volume, which contained 10.0 μl SYBR Green Premix, 0.8 μl of each primer (10 μM), 1.0 μl cDNA template (80 ng/μl), and 7.4 μl ddH_2_O. The PCR parameters were pre-denaturing at 95°C for 30 s, 40 cycles at 95°C for 5 s, and 60°C for 30 s. Melt-curve analyses were carried out using a program with 95°C for 15 s and then a constant growth from 60°C to 95°C with temperature increasing steps of 0.3°C/s. The gene-specific primers were same as those used for semi-quantitative RT-PCR (S2 Table). The watermelon *β-actin* gene (*Cla007792*) was used as an internal reference gene [42]. Each treatment was repeated using three technical replicates. All data from real-time quantitative PCR was calculated for relative expressions following the 2^-ΔΔCt^ method as described by Livak and Schmittgen [43]. The relative expressions values were log_2_ transformed and were displayed in heat map using MeV 4.8.1(http://www.mybiosoftware.com).

## Results

### Identification and characterization of *WRKY* genes

As shown in Table 1, a total of 63 putative *ClWRKY* genes, including 57 full-length *WRKY* genes and 6 WRKY partial homologs, were finally identified in watermelon by HMM (PF03106) and TBLASTN search methods. All of these *ClWRKY* genes could be mapped on the chromosomes from chromosome 1 to 11 and were renamed from *ClWRKY1* to *ClWRKY63* based on their order on the chromosomes (Table 1; S1 Fig). The coding sequences (CDS) of 57 full-length *ClWRKY*s ranges from 366 to 2295 bp and the molecular weight (WM) of them varies from 14049.65 to 82969.81 Da. According to the isoelectric point (pI), 32 *ClWRKY*s were acidic proteins with pI value less than 7.0, and the remaining 25 proteins were basic proteins. According to the instability index, most of *ClWRKY* proteins, with the value of instability index higher than 40.0, were instability, except *ClWRKY13* and *ClWRKY29*. Additionally, the WoLF PSORT prediction showed that 53 *ClWRKY* proteins were localized in nucleus, suggesting ClWRKY proteins play regulatory roles mainly in cell nucleus.

**Table 1:** Watermelon *WRKY* genes and their related information.

Group	Subgroup	Gene name	Gene locus ID	Chromosome	Start site	End site	Genomic (bp)	CDS (bp)	ORF (aa)	MW (Da)	pI	Instability index	Aliphatic index	GRAVY	Subcellular Localization
I		ClWRKY13	Cla015673	2	2511354	2513937	2584	1308	435	47724.69	5.81	38.39	64.41	-0.861	Nucl
		ClWRKY16	Cla013402	2	29711771	29716746	4976	1365	454	49833.11	8.35	65.88	56.48	-0.760	Nucl
		ClWRKY17	Cla008104	3	160231	162530	2300	1765	584	63832.83	7.71	59.59	45.75	-0.911	Nucl
		ClWRKY20	Cla009557	3	15970670	15975906	5237	1944	647	70986.48	4.80	45.09	66.43	-0.647	Nucl
		ClWRKY32	Cla010216	5	31164260	31166896	2637	1482	493	53734.45	6.90	58.42	57.93	-0.865	Nucl
		ClWRKY35	Cla018733	6	21510656	21513842	3187	1542	513	55676.55	6.73	57.67	55.28	-0.965	Nucl
		ClWRKY54	Cla004492	10	4199747	4205383	5637	1728	575	62805.42	6.09	44.89	61.34	-0.766	Nucl
		ClWRKY55	Cla004431	10	4866664	4871403	4740	2295	764	82969.81	6.04	53.77	59.11	-0.702	Nucl
		ClWRKY59	Cla017851	10	26731256	26733477	2222	1452	483	53456.89	8.73	49.16	63.75	-0.742	Nucl
		ClWRKY60	Cla018026	10	28064812	28067485	2674	1428	475	51955.19	6.00	62.22	51.56	-0.785	Nucl
		ClWRKY63	Cla016540	11	22331642	22336203	4562	1515	504	55199.81	8.07	59.28	60.60	-0.777	Nucl
II	a	ClWRKY30	Cla021021	5	24328741	24331742	3002	909	302	33770.91	6.34	41.19	71.36	-0.663	Nucl
	a	ClWRKY49	Cla022362	8	22951835	22953972	2138	924	307	33801.28	8.91	46.09	69.54	-0.582	Nucl
	a	ClWRKY58	Cla017213	10	18648384	18649816	1433	942	313	34349.68	8.70	46.15	64.25	-0.688	Nucl
II	b	ClWRKY4	Cla008346	1	10188973	10192981	4009	1785	594	64179.01	8.15	44.99	58.23	-0.698	Nucl
	b	ClWRKY7	Cla014433	1	30737723	30740093	2371	1872	623	67317.35	6.45	43.91	58.06	-0.752	Nucl
	b	ClWRKY10	Cla007656	2	46121	49115	2995	1638	545	58337.71	6.38	55.83	55.94	-0.707	Nucl
	b	ClWRKY28	Cla013052	5	10364374	10369111	4738	1284	427	47866.14	8.72	57.37	55.08	-1.007	Nucl
	b	ClWRKY37	Cla019127	6	25287085	25291877	4793	1611	536	58252.73	6.74	53.03	60.84	-0.733	Nucl
II	c	ClWRKY1	Cla004938	1	623905	625501	1597	927	308	33142.46	5.14	57.26	64.38	-0.589	Nucl
	c	ClWRKY3	Cla008480	1	8796142	8797069	928	555	184	21088.56	9.27	41.19	54.02	-0.820	Nucl
	c	ClWRKY8	Cla009748	1	32574758	32576313	1556	780	259	28697.44	5.50	47.24	51.58	-0.873	Nucl
	c	ClWRKY12	Cla007761	2	997830	999818	1989	741	246	28331.69	9.17	46.89	57.80	-0.930	Nucl
	c	ClWRKY15	Cla013485	2	28883241	28885653	2413	891	296	33037.24	5.44	54.53	52.70	-0.892	Nucl
	c	ClWRKY21	Cla018197	4	19676671	19679194	2524	993	330	35851.11	9.06	78.76	57.94	-0.616	Nucl
	c	ClWRKY22	Cla021067	5	117031	118615	1585	672	223	25311.98	8.92	47.66	48.43	-0.988	Nucl
	c	ClWRKY24	Cla021203	5	1196965	1198094	1130	372	123	14049.65	7.71	73.55	47.72	-1.120	Mito
	c	ClWRKY26	Cla021806	5	6402958	6404527	1570	1047	348	38019.94	6.00	66.98	55.80	-0.695	Nucl
	c	ClWRKY29	Cla002084	5	18166261	18168850	2590	528	175	19597.98	9.06	32.24	47.89	-0.782	Nucl
	c	ClWRKY34	Cla009235	6	4823071	4824445	1375	936	311	34297.08	6.17	70.09	52.67	-0.746	Nucl
	c	ClWRKY42	Cla014665	7	18910904	18913128	2225	567	188	20902.72	8.19	50.65	69.47	-0.603	Extr
	c	ClWRKY44	Cla010867	7	30588853	30589646	794	423	140	15908.93	9.47	51.33	50.07	-0.836	Cyto
	c	ClWRKY47	Cla021984	8	19308051	19308824	774	462	153	17791.52	5.07	51.12	47.65	-1.098	Nucl
	c	ClWRKY50	Cla015154	9	3914241	3917158	2918	846	281	31889.45	6.55	52.03	58.26	-0.746	Nucl
	c	ClWRKY53	Cla005515	9	34426506	34428311	1806	879	292	33087.73	8.06	59.50	53.77	-0.883	Nucl
	c	ClWRKY57	Cla017345	10	17001468	17004493	3026	918	305	33394.38	6.45	42.48	60.43	-0.567	Nucl
	c	ClWRKY62	Cla003370	11	7658790	7659877	1088	366	121	14204.00	9.46	41.57	49.83	-1.073	Nucl
II	d	ClWRKY6	Cla013967	1	26958654	26959762	1109	894	297	32337.68	9.64	48.87	74.24	-0.528	Nucl
	d	ClWRKY14	Cla006772	2	9026189	9027547	1359	870	289	31279.29	9.58	46.61	72.63	-0.495	Nucl
	d	ClWRKY31	Cla020642	5	28064322	28065905	1584	1143	380	41065.28	9.60	50.51	62.63	-0.592	Nucl
	d	ClWRKY33	Cla009969	5	33154639	33156285	1647	1056	351	37729.73	9.60	53.38	60.40	-0.503	Nucl
	d	ClWRKY36	Cla018870	6	23022662	23024071	1410	837	278	30229.44	9.75	42.23	66.98	-0.560	Nucl
	d	ClWRKY39	Cla006015	7	2332176	2333889	1714	756	251	27970.95	9.67	48.00	63.71	-0.707	Nucl
	d	ClWRKY51	Cla014818	9	6327518	6329225	1708	1071	356	39557.25	9.54	56.93	79.97	-0.478	Nucl
II	e	ClWRKY9	Cla009853	1	33474006	33477082	3077	1455	484	54152.82	5.95	59.70	50.79	-0.998	Nucl
	e	ClWRKY18	Cla019646	3	8018421	8019422	1002	831	276	30132.24	5.15	45.40	55.83	-0.752	Nucl
	e	ClWRKY19	Cla019756	3	9691619	9695338	3720	1203	400	43272.40	5.96	42.58	55.90	-0.732	Nucl
	e	ClWRKY25	Cla021207	5	1219248	1220421	1174	996	331	36795.95	5.78	65.63	55.98	-0.779	Nucl
	e	ClWRKY38	Cla002243	7	1054123	1056739	2617	903	300	31750.79	5.32	59.85	54.37	-0.700	Nucl
	e	ClWRKY56	Cla017355	10	16854843	16856049	1207	954	317	35609.63	4.67	60.56	71.36	-0.733	Extr
III		ClWRKY23	Cla021170	5	925058	926889	1832	1017	338	37697.76	5.09	49.63	68.67	-0.574	Nucl
		ClWRKY27	Cla004233	5	8987121	8988506	1386	1065	354	39061.06	5.35	49.07	62.57	-0.620	Nucl
		ClWRKY40	Cla007306	7	6705089	6711611	6523	978	325	35540.94	6.86	59.33	66.37	-0.478	Nucl
		ClWRKY41	Cla007307	7	6714426	6716677	2252	858	285	31505.05	6.36	44.77	64.14	-0.518	Nucl
		ClWRKY45	Cla010918	7	31145037	31146784	1748	1086	361	40510.40	5.69	55.65	53.77	-0.897	Nucl
		ClWRKY52	Cla015003	9	8487793	8489885	2093	921	306	33831.35	5.46	60.15	58.59	-0.625	Nucl
		ClWRKY61	Cla018059	10	28283762	28284680	919	831	276	31089.43	5.90	54.42	61.88	-0.734	Nucl
NG		ClWRKY2		1	8698894	8699010									
		ClWRKY5		1	25736545	25736845									
		ClWRKY11		2	940572	940688									
		ClWRKY43		7	19000478	19003449									
		ClWRKY46		7	8331842	8332172									
		ClWRKY48		8	19562135	19567420									

NG, no group; CDS, coding sequence; ORF, open reading frame; MW, molecular weight; pI, theoretical isoelectric point; GRAVY, grand average of hydropathicity; Nucl, nuclear; Mito, mitochondrial matrix; Extr, extracellular; Cyto, cytoplasmic.

### Multiple sequence alignment and phylogenetic analysis

**Fig 1:**
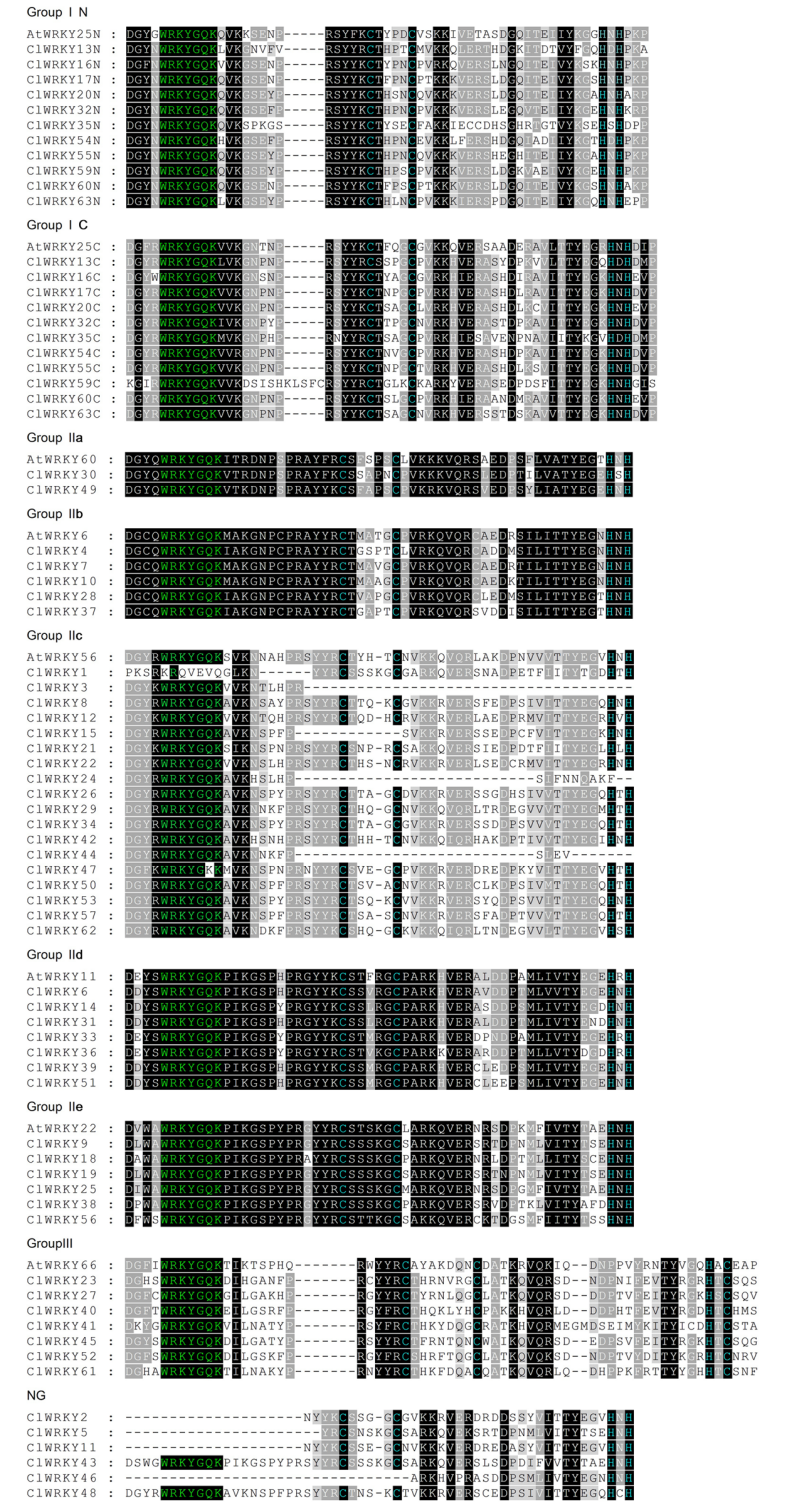
Alignment of 63 *ClWRKY* and 7 *AtWRKY* domain amino acid sequences. Alignment was accomplished using MUSCLE. ‘N’ and ‘C’ indicate the N-terminal and C-terminal WRKY domain of a specific WRKY protein, respectively. The amino acids forming the zinc-finger motif are highlighted in blue, the conserved WRKY amino acid domains is highlighted in green.

The conserved heptapeptide of WRKYGQK is the most obvious structural feature of WRKY proteins, which can interact with the W box to activate target genes [2]. The WRKY domains spanning approximate 60 amino acids were analyzed using multiple sequence alignment (Fig 1). Heptapeptide WRKYGQK in most ClWRKY proteins was highly conserved. However, that in *ClWRKY1* and *ClWRKY47* mutated into KRQVEVQ and WRKYGKK, respectively, and that in *ClWRKY2*, *ClWRKY5*, *ClWRKY11* and *ClWRKY46* was missed. Additionally, identified zinc-finger motifs in some proteins encoded by *ClWRKY3*, *ClWRKY15*, *ClWRKY24*, and *ClWRKY44* were missed.

**Fig 2:**
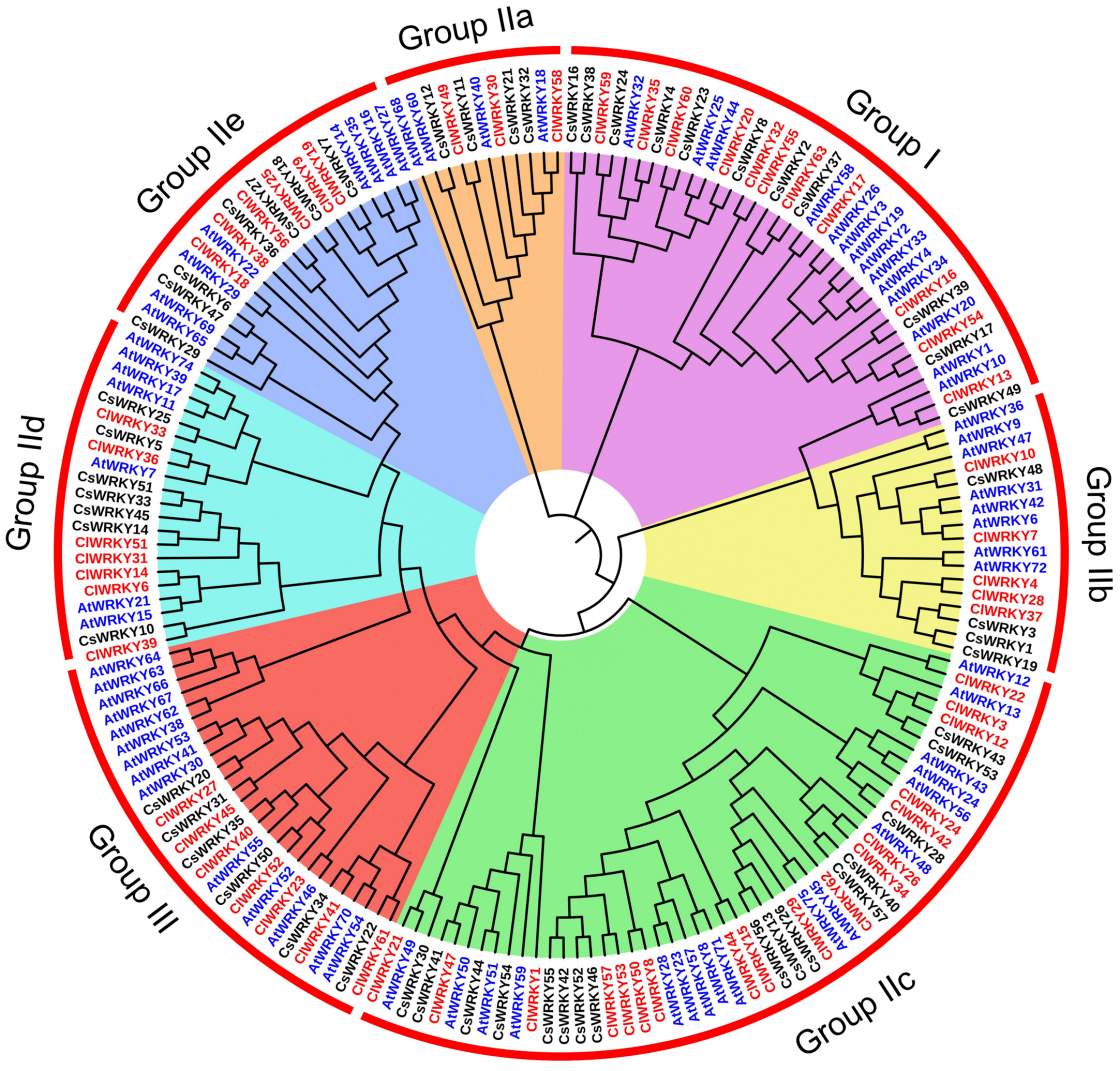
The phylogenetic tree of 184 *WRKY* genes among watermelon (red), *Arabidopsis* (blue) and cucumber (black). The domains clustered into three major groups I, II, III, and five subgroups (a, b, c, d, and e) in group II.

To analyze the evolutionary relationships, a total of 184 *WRKY* genes, including 57 from watermelon, 55 from cucumber, and 72 from *Arabidopsis*, were used to generate a phylogenetic tree (Fig 2). Based on the WRKY domains and the specific zinc-finger motifs, 57 *ClWRKY*s were classified into three groups (I-III) with 11 *ClWRKY*s in group I, 39 in group II, and 7 in group III. The *ClWRKY*s in Group II were further divided into five subgroups (IIa-IIe) with the most genes in Group IIc. Obviously, proteins in group I contained two WRKY domains located at both the N-terminus and C-terminus and the zinc finger motif of C_2_H_2_ type at N-terminus and C-terminus was C-X_4_-C_22_-H-X-H and C-X_4_-C_23_-H-X-H, respectively (Fig 1). By contrast group I, group II and group III genes had only one WRKY domain. Proteins in group IIa, IIb, IId and IIe contained a zinc finger motif of C-X_5_-C_23_-H-X-H, whereas those in group IIc contained a zinc finger motif of C-X_4_-C_23_-H-X-H at C-terminus, except *ClWRKY1*. Group III proteins contained a C_2_HC zinc-finger motif of C-X_7_-C-X_23_-H-X-C at C-terminus. In addition, sequence comparisons and phylogenetic analyses showed that WRKY proteins from three species appeared scattered across the branches of the evolutionary tree, implying that they experienced duplications after the lineages diverged. Meanwhile, a total of 42 *WRKY* genes from cucumber and watermelon were clustered as 21 pairs (Fig 2), indicating that they were the orthologous WRKY domains from the same lineage.

### Gene structure and conserved motifs analysis

**Fig 3:**
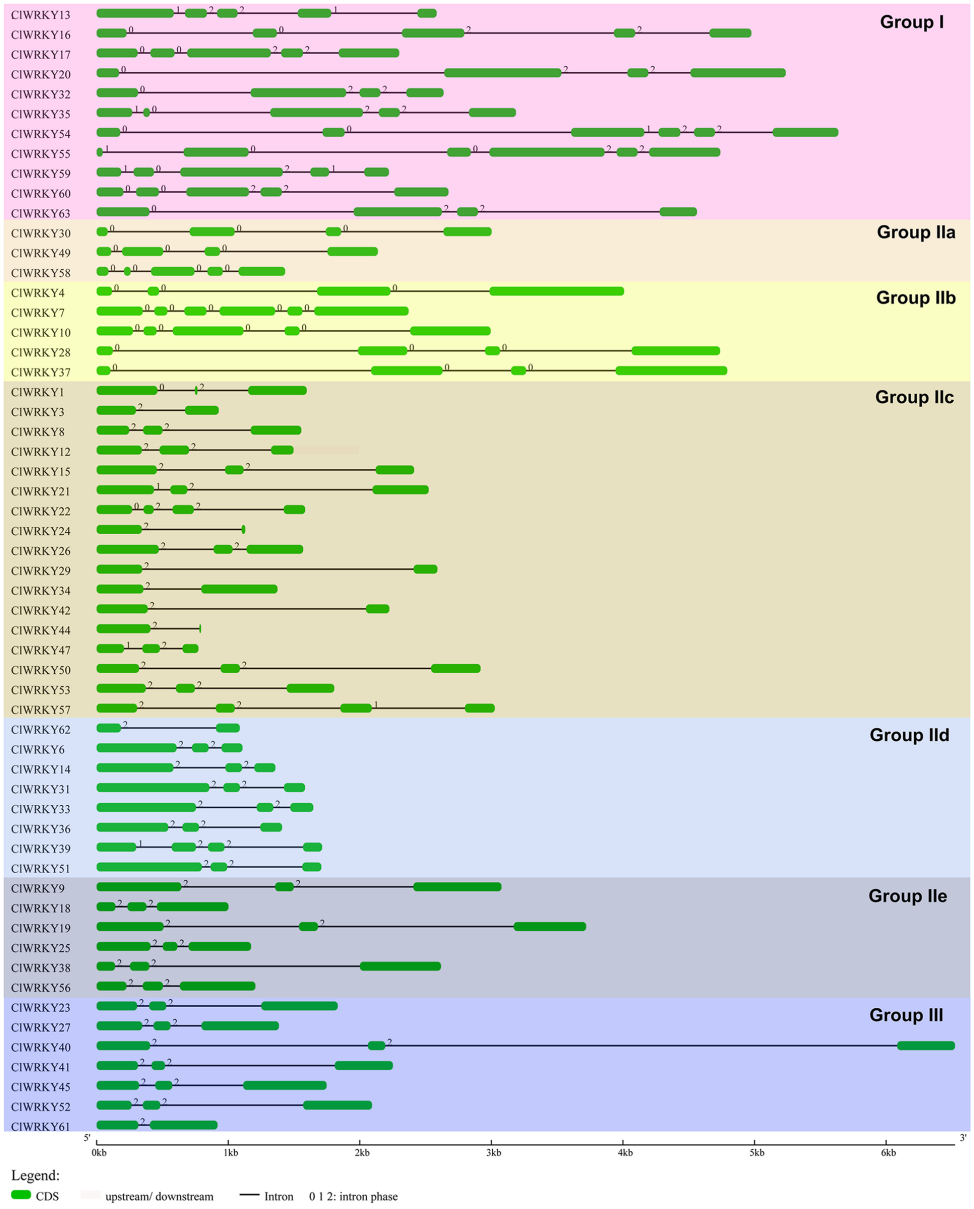
Illustration of the gene structure of 57 *ClWRKY* transcription factors. Genes were separated into their respective groups with different colors. Exons were shown using green round-corner rectangle while introns were shown using black solid lines (5′-3′).

The exon-intron analysis was performed to obtain a better insight into the structure of *ClWRKY* genes, which exhibited relatively smaller variation in numbers of exons and introns. As shown in Fig 3, 57 *ClWRKY* genes had two to six exons. Among them, 27 *ClWRKY*s had three exons, followed by eleven *ClWRKY*s with four exons, eight *ClWRKY*s with two exons, eight *ClWRKY*s with five exons, and three *ClWRKY*s with six exons. These divergences suggested that both exon gain and loss was occurred during the evolution of the *WRKY* gene family. *ClWRKY* genes in the same group usually seemed to have similar exon-intron structures. For instance, six genes from seven members in Group III had three exons. In comparison to *ClWRKY*s in group II and group III, those in group I had more exons, ranging from four to six. These findings provided an additional foundation to support the classification of *ClWRKY*s.

**Fig 4:**
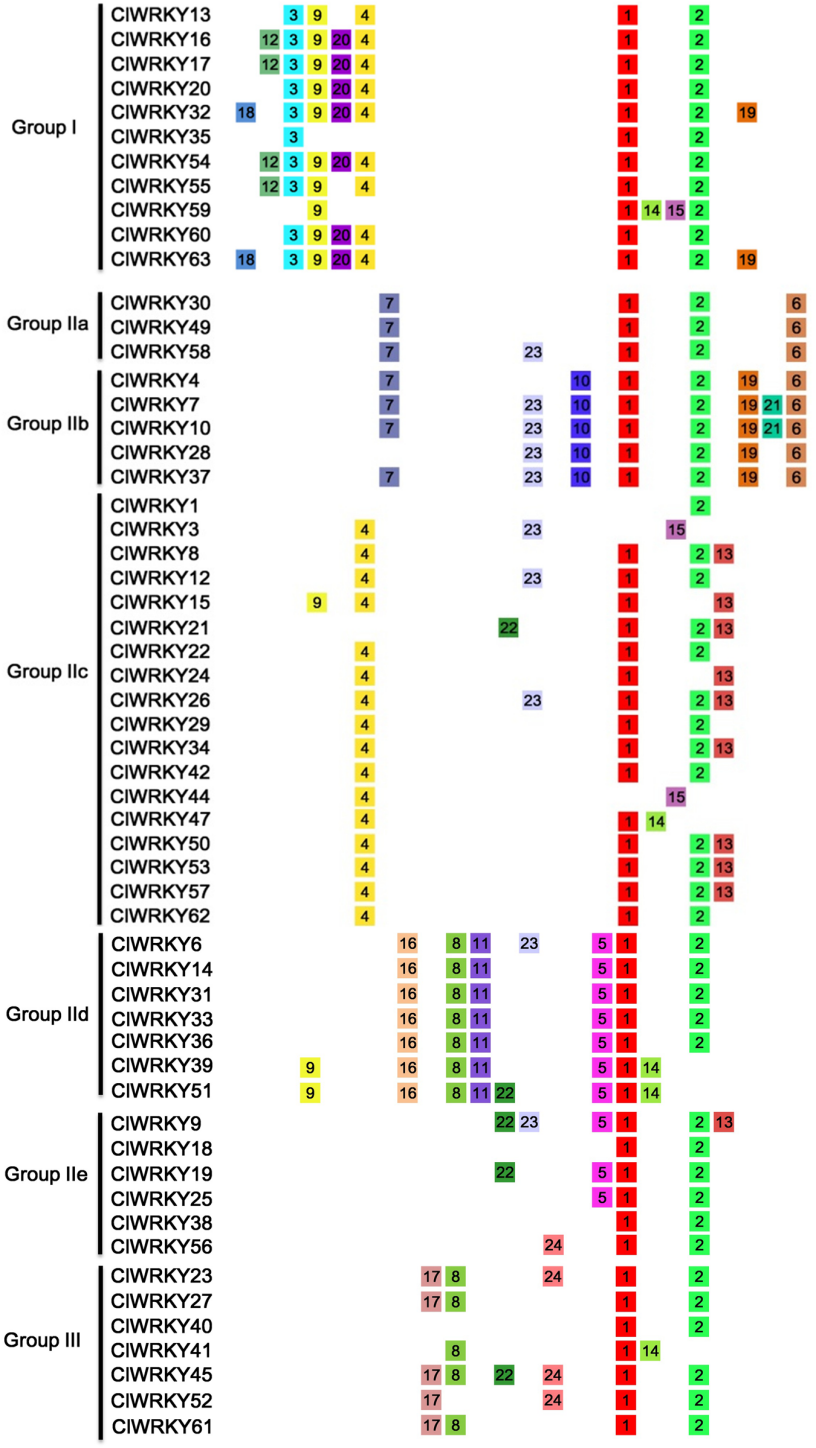
Schematic representation of 24 conserved motifs in watermelon WRKY proteins. Conserved motifs were named as motif 1 to motif 24, and different motif was shown as colored boxes with their names in the center of the boxes. The colored boxes were ordered manually according to the results of the MEME analysis. The length of each box in the figure does not represent the actual motif size.

We further searched for the conserved motifs in 57 *ClWRKY* proteins using MEME program (Fig 4). In total, 24 conserved motifs named as motif 1 to motif 24 were identified and the details of the 24 putative motifs were listed in S3 Table. Motif 1 and 2 were found in most of *ClWRKY* genes. Motifs 1, 3, and 15 contained a WRKYGQK sequence. As expected, most ClWRKY proteins in the same group or subgroup possessed similar motifs, suggesting their functional equivalency. For example, motifs 1, 2, 3, 4, and 9 were present in group I proteins, which contained two WRKY domains, whereas group III proteins possessed motifs 1, 2, 8, and 17.

### Gene duplication and synteny analysis

**Fig 5:**
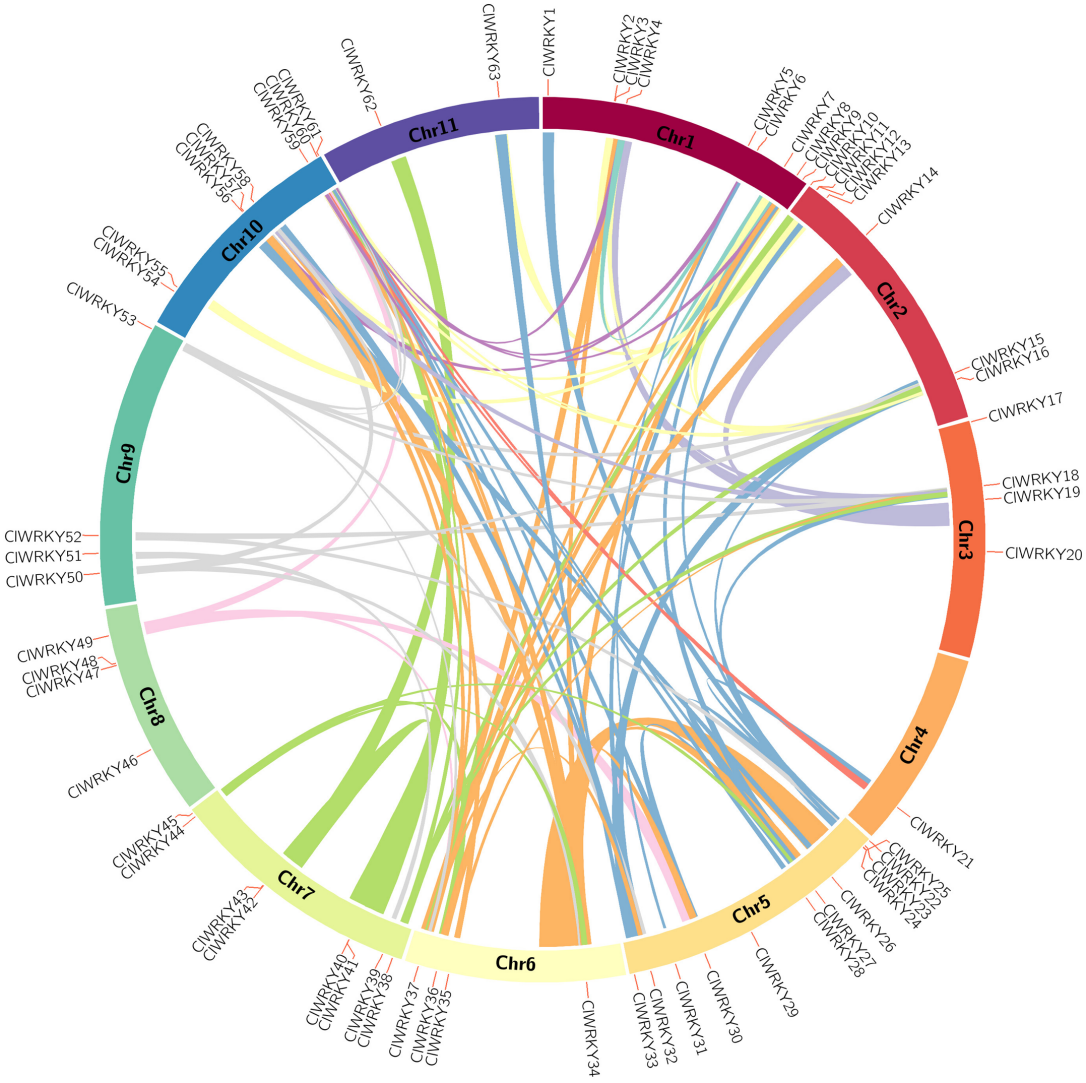
Synteny analysis of watermelon *WRKY* genes. Chromosomes 1–11 were shown with different colors and in a circular form. Colored curves indicated the details of syntenic regions in watermelon genome.

Currently, we evaluated the gene duplication events of *ClWRKY* genes using MCScanX program. Ninety-four syntenic relations of *ClWRKY*s were identified as duplication events in watermelon genome (Fig 5; S4 Table), and 45 *ClWRKY* genes were located within syntenic blocks on all watermelon chromosomes. Chromosome 1, 5 and 10 had more duplication regions and that could partly explain the larger numbers of *ClWRKY* genes located on these three chromosomes. Gene duplication events are defined as either tandem duplications, with two or more genes located on the same chromosome, or segmental duplications, with duplicated genes present on different chromosomes [44]. There were 11 *ClWRKY* genes clustered into 6 tandem duplication event regions on watermelon chromosome 1 (*ClWRKY4*/*ClWRKY6* and *ClWRKY7*/*ClWRKY8*), 5 (*ClWRKY23*/*ClWRKY27* and *ClWRKY27*/*ClWRKY30*), 7 (*ClWRKY40*/*ClWRKY42*), and 10 (*ClWRKY59*/*ClWRKY60*). The other 88 syntenic relations of *ClWRKY* genes were confirmed as segmental duplications, suggesting that most *ClWRKY* genes were possibly generated by gene segmental duplication.

**Fig 6:**
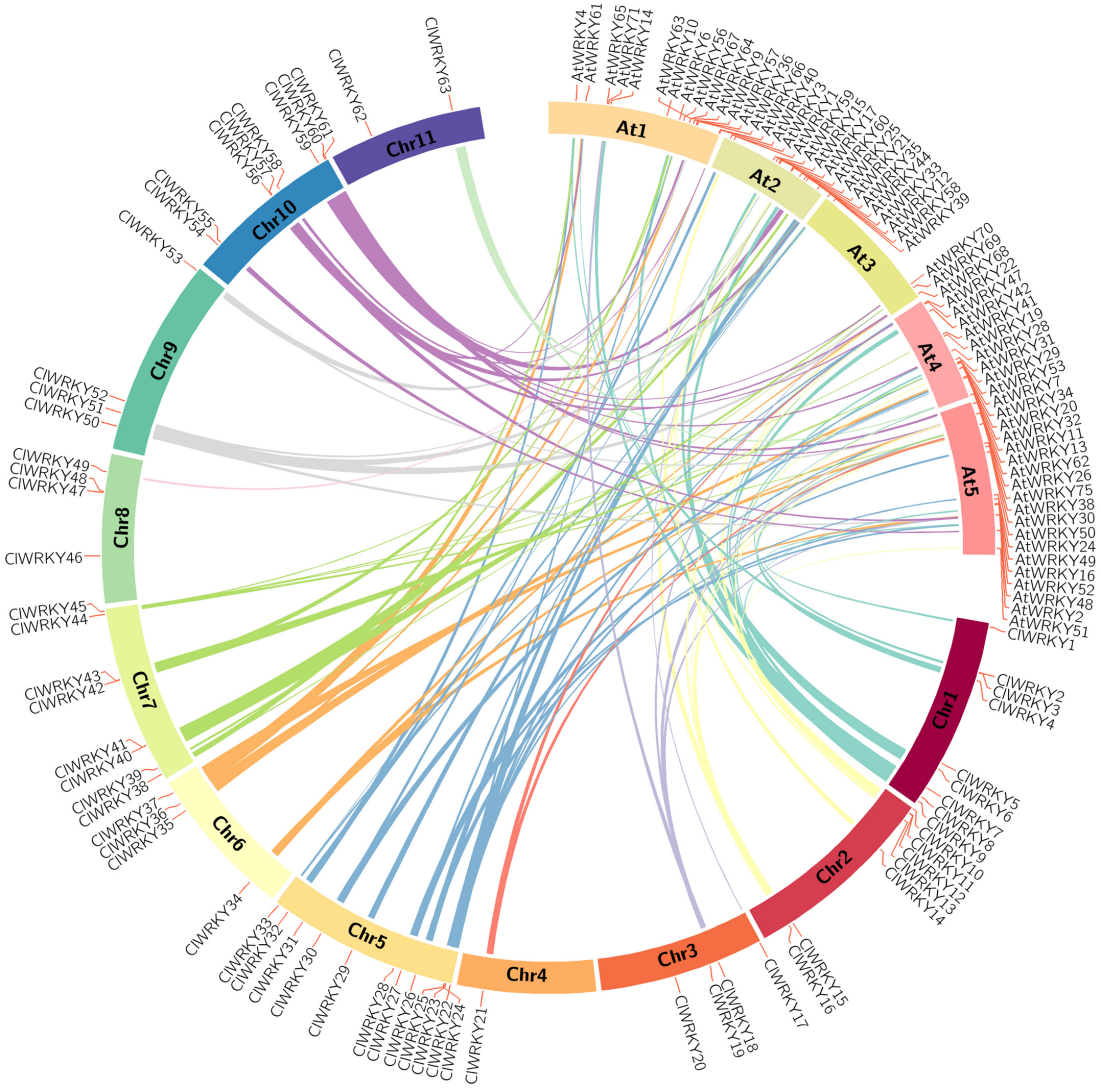
Synteny analysis between watermelon and *Arabidopsis WRKY* genes. The chromosomes of watermelon and *Arabidopsis* are depicted as a circle. Colored curves denote the details of syntenic regions between watermelon and *Arabidopsis WRKY* genes.

*Arabidopsis* was widely used as a model system for plant *WRKY* TFs research [45]. In order to further explore evolutionary and functionality connections between watermelon and *Arabidopsis WRKY* genes, a synteny analysis was performed. A total of 103 pairs of syntenic relations were identified, including 51 *AtWRKY* genes and 45 *ClWRKY* genes (Fig 6; S5 Table). Out of these genes, 13 *AtWRKY* genes and 18 *ClWRKY* genes were found to be associated with at least three synteny events, and three *ClWRKY* genes (*ClWRKY8*, *ClWRKY18* and *ClWRKY23*) were involved in six synteny events. A lot of synteny events indicate that numerous *WRKY* genes existed before the divergence of the *Arabidopsis* and watermelon.

### Expression profiles of the *ClWRKY* genes in different tissues

**Fig 7:**
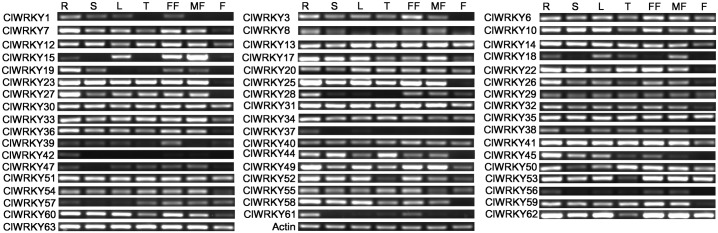
Expression profiles of *ClWRKY* genes in various tissues of ‘Y34’ by semi-quantitative RT-PCR analyses. *β-actin* gene was used as the internal control. Six amplified bands from left to right for each *WRKY* gene represent amplified products from R: root; S: stem; L: leaf; T: tendril; FF: female flower; MF: male flower and F: fruit.

By means of semi-quantitative observation, we analyzed the expression profiles of 57 full-length *ClWRKY* genes in seven different tissues including roots, stems, leaves, tendrils, fruit, male flowers, and female flowers under normal growth conditions. Then, the gene-specific primers of 52 *ClWRKY*s were successfully found in our study (S2 Table). The results showed that 52 *ClWRKY*s genes were detected in at least one of the seven tested tissues (Fig 7). Among them, 17 (33%) *ClWRKY* genes were expressed in all tested tissues. The other genes were detected in only one or several tissues. For instance, *ClWRKY37* and *ClWRKY42* were found only in roots, whereas *ClWRKY15* was preferential accumulation in male flower. Some genes such as *ClWRKY61* and *ClWRKY56* were not observed in any tested tissue possibly due to their expression in other tissues or too low expression level (below detection limit via semi-quantitative observation).

### Expression patterns of the *ClWRKY* genes under various abiotic stresses

**Fig 8:**
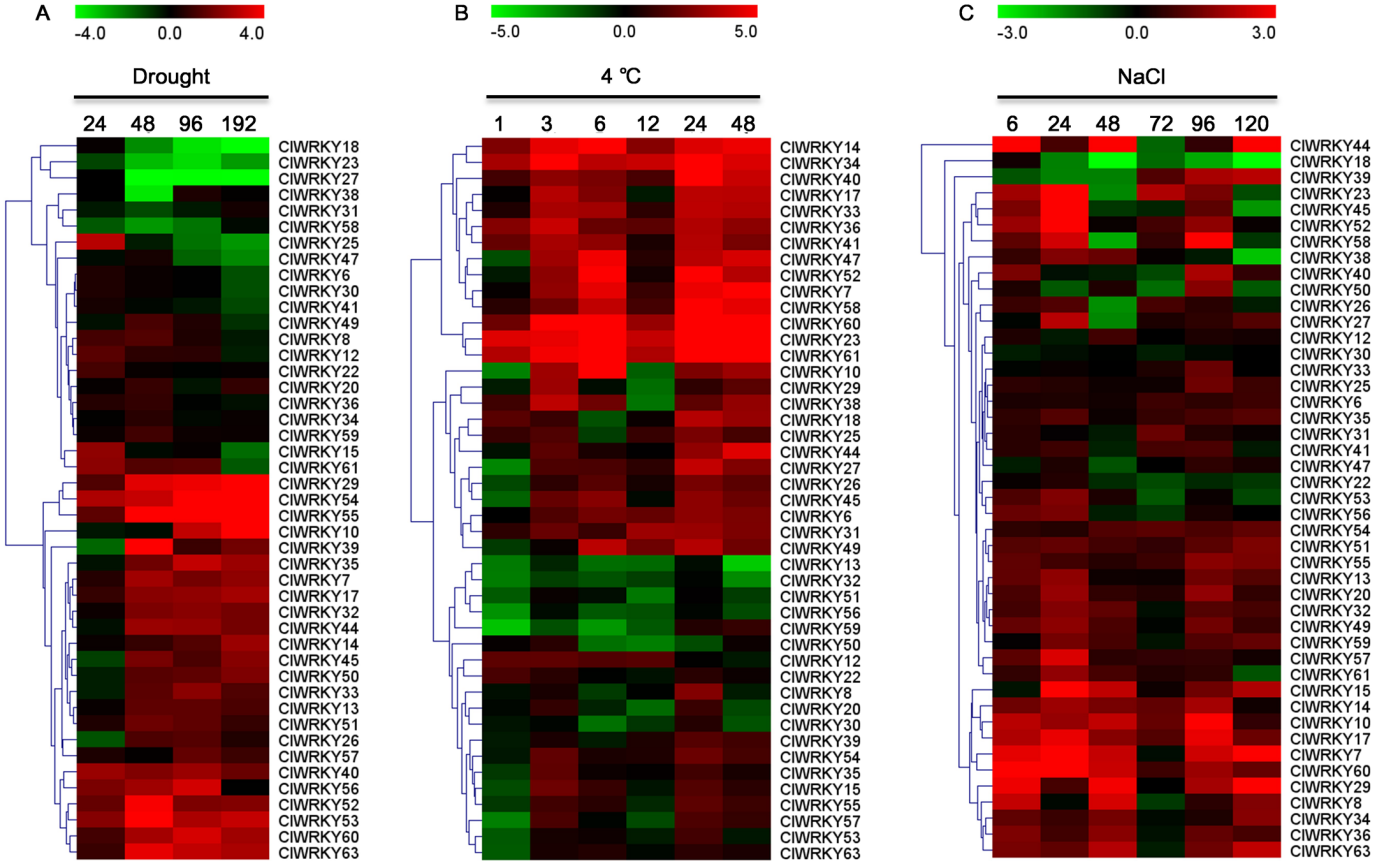
Expression patterns of *ClWRKY* genes under abiotic stresses by qRT-PCR. The abiotic stresses used for expression patterns are indicated at the top. Scale bars on the top of each heat map represent log_2_-transformed (Treatment/Control) values, with red as increased expressed level and green as decreased expressed level. (A) Expression of *ClWRKY* genes under drought stress, and 24, 48, 96, and 192 indicate hours after treatment. (B) Expression of *ClWRKY* genes under 4°C treatment, and 1, 3, 6, 12, 24, and 48 indicate hours after treatment. (C) Expression of *ClWRKY* genes under salt stress treatment, and 6, 24, 48, 72, 96, and 120 indicate hours after treatment.

To investigate the potential roles of *ClWRKY* genes in response to abiotic stresses, we analyzed their dynamic response after exposure to drought, cold, and salinity stresses using qRT-PCR. *ClWRKY* genes exhibited different expression patterns in response to different stresses (Fig 8). Drought treatment continuously induced the expression of about half of the detected *ClWRKY* genes but reduced the expression of four *ClWRKY* genes including *ClWRKY18*, *ClWRKY23*, *ClWRKY27* and *ClWRKY58*. The expression of *ClWRKY15*, *ClWRKY25* and *ClWRKY61* transiently increased at 24 h but decreased at 192 h after drought treatment. Additionally, 11 *ClWRKY*s such as *ClWRKY12*, *ClWRKY34*, and *ClWRKY41* slightly induced or reduced at the earlier or later period of drought stress. Similarly, cold stress (4°C) continuously up-regulated and down-regulated about half of detected *ClWRKY*s and four *ClWRKY* genes (*ClWRKY13*, *ClWRKY32*, *ClWRKY51* and *ClWRKY56*), respectively. Several *ClWRKY*s such as *ClWRKY27* showed a transient down-regulation at 1 h but then continuously up-regulated with the advancement of cold stress. Most of *ClWRKY* genes were up-regulated but only *ClWRKY18* were down-regulated by the NaCl treatment. Some *ClWRKY*s such as *ClWRKY58* showed a transient down-regulation at the median or later period of salt stress. Notably, the expressions of *ClWRKY14* and *ClWRKY60* were up-regulated by all stresses, suggesting that these two genes may play a core role in plant tolerance to diverse abiotic stresses.

### Expression patterns of the *ClWRKY* genes to hormone treatments

**Fig 9:**
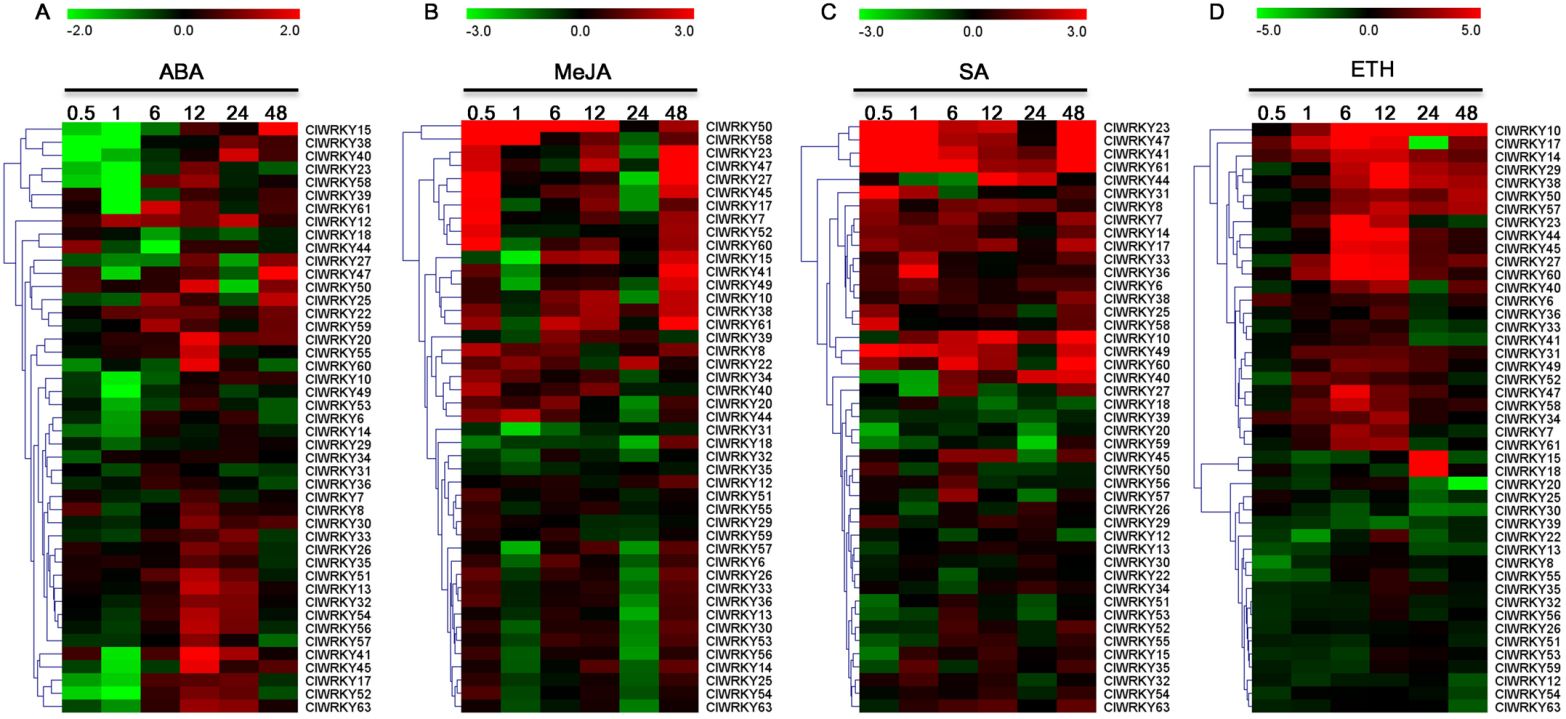
Expression patterns of *ClWRKY* genes under exogenous hormone treatments. Scale bars on the top of each heat map represent log_2_-transformed (Treatment/Control) values. The hormone treatments including abscisic acid (ABA), methyl jasmonic acid (MeJA), salicylic acid (SA) and ethephon (ETH) are shown as heatmaps (A), (B), (C) and (D), respectively. 0.5, 1, 6, 12, 24 and 48 indicate hours after treatment.

Numerous evidences have indicated that *WRKY* TFs are involved in signal pathways of various plant hormones such as ABA, MeJA, SA, and ETH [46]. As shown in Fig 9, ABA treatment induced both up- and down-regulation of almost all *ClWRKY*s at different time points. Most of *ClWRKY*s were down-regulated or remained unchanged at 0.5 and 1 h but ABA application up-regulated their expression at 12 h and 24 h. Similarly, many of *ClWRKY* genes were both up-regulated and down-regulated at different periods by MeJA, as well as ABA treatment. Most of *ClWRKY*s were induced or remained unchanged at 0.5, 6, 12, and 48 h but MeJA treatment reduced their expression at 24 h. Being different with ABA and MeJA, SA application induced continuous up-regulation of 12 *ClWRKY*s and down-regulation of three *ClWRKY*s. The other *ClWRKY*s showed different expression patterns at different time-points after SA treatment. In response to ETH treatment, about a half of *ClWRKY* genes showed continuous up-expression from 1 to 48 h, with some exceptions showing down-expression at 24 h and 48 h. ETH treatment down-regulated expression of the other *ClWRKY*s. These results indicate that *ClWRKY* genes are positively or negatively involved in regulatory pathways of plant hormones and thus play important roles in plant growth, development, and defense against environmental stresses.

## Discussion

### Annotation and characterization of *WRKY* genes in watermelon genome

Due to the important roles of *WRKY* transcriptional factors in plant growth, development, and response to stresses, a large number of WRKY families have been identified in diverse plant species (S6 Table). However, there is dearth of information about *WRKY* genes in watermelon. Here, we identified a total of 63 putative *ClWRKY* genes in watermelon. In addition to 57 full-length *ClWRKY* genes, 6 *ClWRKY* partial homologs (*ClWRKY2*, *ClWRKY5*, *ClWRKY11*, *ClWRKY43*, *ClWRKY46* and *ClWRKY48*) were found using TBLASTN method with in-house Perl scripts. This operation ensured that *WRKY* genes were complete in watermelon genome, although no particular annotation information was available for these 6 WRKY partial homologs. Moreover, our further analysis revealed the properties (i.e. amino acid length, molecular weight, isoelectric point, and instability) and subcellular location of ClWRKY proteins.

According to the classification of *WRKY* genes in *Arabidopsis* [2], 57 full-length *ClWRKY* genes were also classified into three groups (I-III) and five subgroups (IIa-IIe) of group II based on their conserved WRKY domain and zinc-finger motif. The size of *ClWRKY*s in each group is similar to that of *CsWRKY*s in cucumber [47], indicating a similar evolutionary pattern between watermelon and cucumber (Fig 2). However, the size of the *ClWRKY* gene family (63) is small compared to that of model plants such as *Arabidopsis* (72) and rice (102). The differences in the number of *WRKY* genes in group III are the primary cause of the diverse sizes of *WRKY* gene families (S6 Table). Group III *WRKY* genes have been described as a newly defined and the most dynamic group with a large number of duplications events [7]. Therefore, group-III *WRKY* genes may play important roles in plant evolution.

The number of exons in *ClWRKY* genes varied from two to six (Fig 3), and the exon-intron structural diversification might be caused by the rearrangement and fusions of different chromosome fragments [44, 48]. This finding provided an additional foundation to support the classification of *ClWRKY*s and a way to find out which group *WRKY* genes might be of a more ancient origin [8]. Moreover, we should pay attention to the roles of special motif in only one group, such as motif 16 in group IId proteins and motif 17 in group III proteins (Fig 4), which may possess some hitherto uncharacterized roles.

Multiple sequence alignments revealed that WRKYGQK sequence was highly conserved in most of *ClWRKY* TFs. The WRKYGQK sequence was considered to be important for recognizing and binding to W box elements in the promoter of target genes [3]. Previous studies have reported a number of variants of the WRKYGQK sequence in diverse plant species and proteins with these variants may recognize the other binding elements other than the W box element [7, 49]. In watermelon *WRKY* genes, we found two variants of the WRKY domain: WRKYGKK in *ClWRKY1* and KRQVEVQ in *ClWRKY47* (Fig 2). WRKYGKK sequence was the most common variant in many plants and proteins with this sequence instead of WRKYGQK could bind specifically to WK box (TTTTCCAC) [50, 51]. Strikingly, variant KRQVEVQ sequence was found only in watermelon (*ClWRKY1*). Variations of WRKYGQK motif might change DNA binding specificities of downstream target genes, and thus it would be interesting to validate the biological functions of *ClWRKY1* and *ClWRKY47*. Moreover, deletion events of the zinc-finger motif occurred in four *ClWRKY* genes (*ClWRKY3*, *ClWRKY15*, *ClWRKY24* and *ClWRKY44*) in group IIc. The replacement or deletion of the zinc-finger motif might lead to the evolution and classification of *WRKY* genes [44, 52]. However, it remains largely unknown whether the deletions of zinc-finger motif influence the function and the expression patterns of *WRKY* genes.

### Expansion and synteny of *ClWRKY* genes

Gene duplications played a crucial role in genomic rapid expansions and evolution of gene families [53]. A number of evidence pointed that duplication and expansion events happened in plant *WRKY* genes [44, 54, 55]. Here, 6 tandem duplication event regions and 88 segmental duplications event regions of *ClWRKY* genes were confirmed in watermelon chromosome (Fig 5; S4 Table), suggesting that low-tandem and high-segmental duplications events existed in *ClWRKY* genes family. This finding is in agreement with that in *Arabidopsis* and *Populus trichocarpa* [52, 54], but incongruent with that in rice [21], which could be due to a large scale artificial selection and the differences of life cycle between rice and watermelon. Interestingly, three *ClWRKY* genes (*ClWRKY23*, *ClWRKY27* and *ClWRKY40*) with tandem duplication were belonged to group III genes, implying that a different pattern of duplication may cause smaller size of group III *WRKY* genes in watermelon.

Moreover, most of *ClWRKY* genes were found in syntenic genomic regions of *Arabidopsis* (Fig 6; S5 Table). The large numbers of gene duplication events between watermelon and *Arabidopsis* will help us to understand the functions of *ClWRKY* genes. For example, over-expression of *AtWRKY57* in rice improves drought and salt tolerance [56]. Moreover, *AtWRKY70* is involved in brassinosteroids (BRs)-regulated growth and negatively affects drought responses [57]. Meanwhile, *ClWRKY60* and *ClWRKY61* were in the synteny region with these well-known *AtWRKY* genes, predicting a similar functional mechanism of *ClWRKY60* and *ClWRKY61* in drought resistance in watermelon. Nevertheless, gene functional study is required to better predict their roles.

### Possible roles of *ClWRKY* genes in normal and stress conditions

Results from numerous studies demonstrate that *WRKY* transcription factors play very critical role in different tissues to regulate plant growth and developmental processes [3]. For instance, virus-induced silencing of *GmWRKY58* and *GmWRKY76* in soybean causes severe stunted growth with reduced leaf size and plant stature [58], and overexpression of the *OsWRKY31* could reduce lateral root formation and elongation [10]. In our study, *ClWRKY13*, *ClWRKY31*, *ClWRKY51* and *ClWRKY63* showed higher expression levels in all tested tissues (Fig 7). Among them, *ClWRKY13* and *ClWRKY63* were orthologous genes of *CsWRKY49* and *CsWRKY37*, respectively, implying that these genes play key roles in the whole-plant growth and development [21, 47]. Furthermore, some *ClWRKY* genes were specifically expressed in a certain tested tissue. As shown in Fig 7, *ClWRKY15* were preferentially expressed on male flower, suggesting that *ClWRKY15* might play important roles during development of male reproductive organs. In addition, *ClWRKY* genes with low expression level in all tested tissues might be expressed specifically in other tissues such as seeds, or could be induced under environment stimuli. For example, *ClWRKY56* and *ClWRKY61* were not detected in leaves under normal condition, but they were induced by abiotic stresses (Fig 7), suggesting the involvement of these genes in stress signaling.

Increasing evidence show that WRKY proteins in various plant species are involved in the response plants to various abiotic stresses such as drought, cold, and salt [16, 59]. In *Arabidopsis*, rice, and cucumber, at least 20, 54, and 23 *WRKY* genes were identified in response to diverse abiotic stress, respectively [17, 21, 47]. The responses of *ClWRKY*s to abiotic stress and plant hormones treatments can provide useful clues for dissecting the potential roles of *WRKY* genes in watermelon. In this study, we found that most of *ClWRKY*s positively or negatively responded to drought, cold, and salt stresses, and their responses altered with the degree of stresses. These results provided a useful reference for functional verification of *ClWRKY*s under environmental stresses. Additionally, the expression patterns of *ClWRKY*s in watermelon responses to abiotic stresses differed greatly from those in other plant species such as cucumber and pear [47, 60], suggesting that there were different gene duplication and evolution ways among different plant species.

Orthologous genes are generally supposed to retain equivalent functions in different species and to share other key properties [60]. The comparative analysis of *ClWRKY* genes with their homologous genes in other plant species helped to predict the potential functions of WRKY proteins in watermelon. In cucumber, *CsWRKY46* can be induced by various stresses and its overexpression conferred cold tolerance to transgenic plants by positively regulating signaling pathway [47, 61]. As its orthologous genes, *ClWRKY8*, *ClWRKY34* and *ClWRKY53* also showed significant up-regulation under salt, cold, and drought stress, respectively (Fig 8), these genes may play similar roles with *CsWRKY46* in stress response. Ding et al. [62] demonstrated that *AtWRKY46* played dual roles in regulating plant responses to drought and salt stresses. Its orthologous gene *ClWRKY23* also was found to be up-regulated under drought and salt stresses. *AtWRKY25* in *Arabidopsis* has been known to respond to both heat and salt treatments [63]. Similarly, its orthologous gene *ClWRKY60* is obviously and continuously induced by multiple abiotic stresses. These results imply that a single *WRKY* gene may play various regulatory roles in response to stresses. In addition, *AtWRKY70* and *AtWRKY54* co-operate as negative regulators for stomatal closure and thus regulate osmotic stress tolerance in *Arabidopsis* [20]. The negative responses of their orthologous genes *ClWRKY41* and *ClWRKY61* at the end of drought treatment imply they can play negative regulatory roles at the later period of drought.

Given that plant hormones such as ABA, SA, JA, and ETH, play critical roles in regulating plant growth, development, and defense against various abiotic and biotic stresses [17, 64, 65], a number of *WRKY* genes have been demonstrated to be involved in diverse plant hormone signal pathways [20, 23]. Ding et al. [66] revealed that the expression of *AtWRKY46* was repressed by ABA signal, but induced by an ABA-independent signal under osmotic and salt stress, as well as *AtWRKY54*, *AtWRKY70*, and *CsWRKY46* [20, 61]. As homologous of these well-known *WRKY* genes, *ClWRKY60*, *ClWRKY23*, *ClWRKY41* and *ClWRKY61* expressions in watermelon were up-regulated by drought stress (Fig 8), but were down-regulated by ABA at 0.5 or 1 h after treatment (Fig 9). Thus, these four *ClWRKY* genes may regulate watermelon response to drought stress via interacting with ABA signaling. In addition, Cho et al. [67] and Yang et al. [68] found that *ClWRKY70* (same as *ClWRKY41* in this study) and *ClWRKY1* (same as *ClWRKY17* in this study) play a positive regulatory role in plant resistance against pathogen attack. In our study, *ClWRKY41* and *ClWRKY17* were significantly up-regulated by SA treatment, implying that these two genes were involved in SA-mediated signaling pathways in plant defense responses. These results indicate that the regulatory mechanism of *WRKY* proteins under abiotic stresses is complex and the functional dissection of *WRKY* genes in signaling pathways and stress responses will be an important research topic for the future.

## Conclusions

In this study, we identified watermelon *WRKY* family genes and analyzed their expression patterns for the first time. A total of 63 putative *ClWRKY* genes in watermelon were obtained using comprehensive computational approaches. All 63 *ClWRKY* genes located on 11 chromosomes were classified into three major groups (I-III) and five subgroups (IIa-IIe) in group II. *ClWRKY*s may play multiple regulatory roles based on their varied structures with conserved or varied WRKY domain and different motifs. The expression of *ClWRKY*s in different tissues indicated that they were involved in various tissue growth and development. Furthermore, the diverse responses of *ClWRKY*s to abiotic stresses suggested that they positively or negatively participated in plant tolerance against drought, salt, or cold stress. Altered expression patterns of *ClWRKY*s by phytohormones such as, ABA, SA, MeJA, and ETH, implied the existence of complex crosstalks between *ClWRKY*s and plant hormone signals in regulating plant physiological and biological processes. Therefore, our findings provide valuable clues for further research on the function and regulatory mechanisms of *ClWRKY* TFs in watermelon growth, development, and adaption to environmental stresses.

## Supporting information

S1 Table Full-length protein sequence of *WRKY* genes from watermelon, *Arabidopsis*, and cucumber. (XLSX)

S2 Table The primers of *WRKY* genes in watermelon. (XLSX)

S3 Table The details of the 24 putative motifs of watermelon *WRKY* genes. (DOCX)

S4 Table The synteny regions between watermelon *WRKY* genes. (XLSX)

S5 Table The synteny regions between watermelon and *Arabidopsis WRKY* genes. (XLSX)

S6 Table Number of *WRKY* TF gene family members present in major crop plants and horticultural plants. (DOCX)

S7 Table The relative expression of watermelon *WRKY* genes via qRT-PCR in response to various abiotic stresses and hormone treatments. (XLSX)

S1 Fig Genetic map position on watermelon chromosomes of *WRKY* gene family. The numbers indicate the start site of *WRKY* genes located on chromosomes.
(TIF)
