# Supplementary material for: Identification and expression analyses of WRKY genes reveal their involvement in growth and abiotic stress response in watermelon (Citrullus lanatus)
Source: PLoS One. 2018 Jan 16;13(1):e0191308. doi: 10.1371/journal.pone.0191308 (PMC5770075; doi:10.1371/journal.pone.0191308)
Supplement: S3 Table — (DOCX) [file pone.0191308.s003.docx]

**Table S3. The details of the 24 putative motifs of watermelon *WRKY* genes.**

| **Motif** | **E-value** | **Sites** | **Width** | **Logo** |
| --- | --- | --- | --- | --- |
| **Motif 1** | 1.7e-1161 | 54 | 29 | 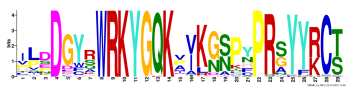 |
| **Motif 2** | 1.4e-851 | 49 | 40 | 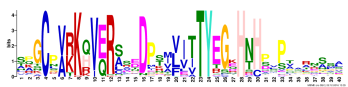 |
| **Motif 3** | 7.2e-315 | 10 | 50 | 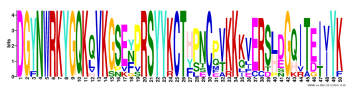 |
| **Motif 4** | 6.0e-153 | 25 | 21 | 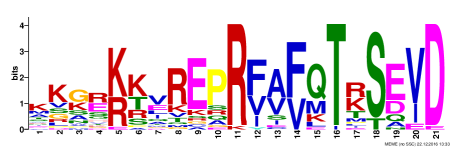 |
| **Motif 5** | 2.5e-059 | 10 | 21 | 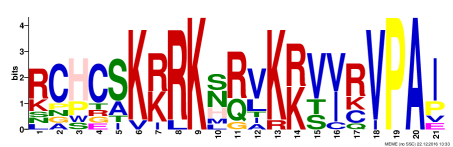 |
| **Motif 6** | 2.6e-036 | 8 | 28 | 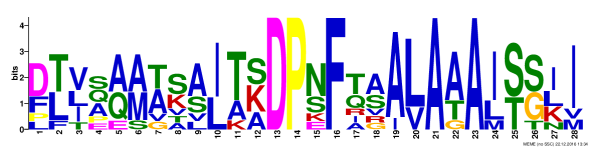 |
| **Motif 7** | 1.5e-037 | 7 | 39 | 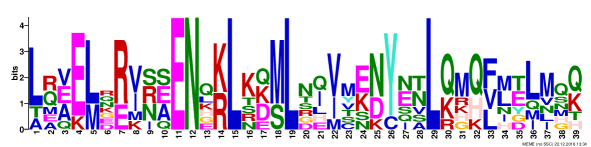 |
| **Motif 8** | 9.5e-029 | 12 | 21 | 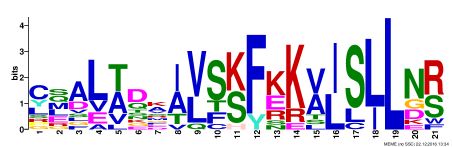 |
| **Motif 9** | 1.5e-026 | 13 | 14 | 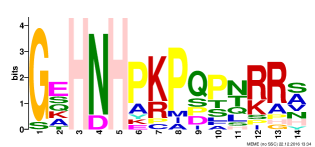 |
| **Motif 10** | 2.6e-013 | 5 | 15 | 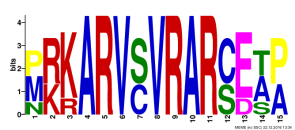 |
| **Motif 11** | 3.1e-013 | 7 | 10 | 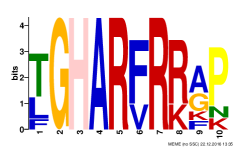 |
| **Motif 12** | 3.7e-013 | 4 | 34 | 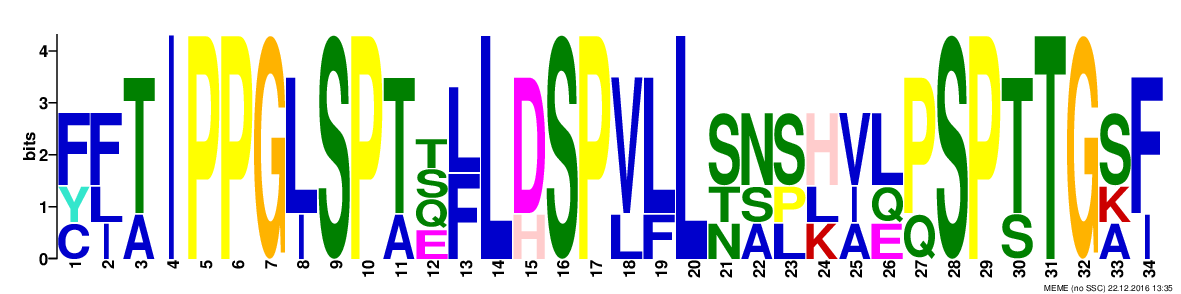 |
| **Motif 13** | 8.1e-010 | 10 | 11 | 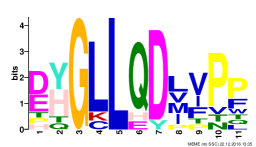 |
| **Motif 14** | 4.1e-009 | 5 | 21 | 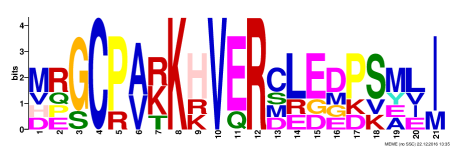 |
| **Motif 15** | 7.8e-008 | 3 | 18 | 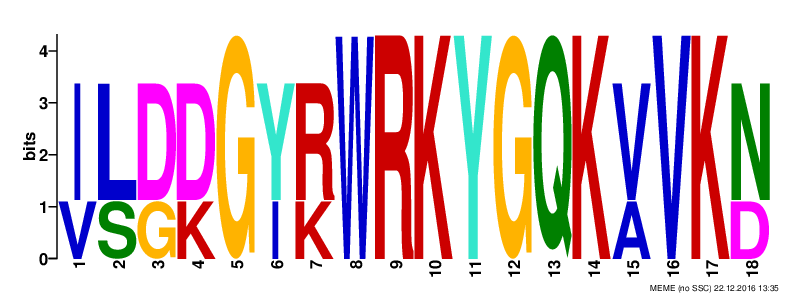 |
| **Motif 16** | 1.3e-007 | 7 | 21 | 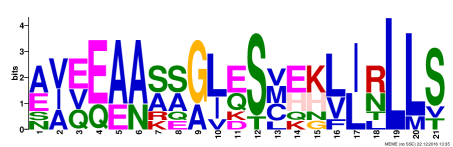 |
| **Motif 17** | 2.1e-007 | 5 | 25 | 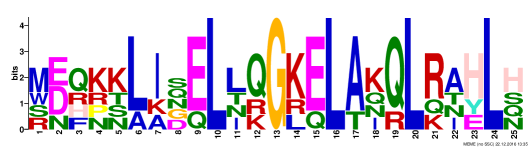 |
| **Motif 18** | 1.4e-005 | 2 | 35 | 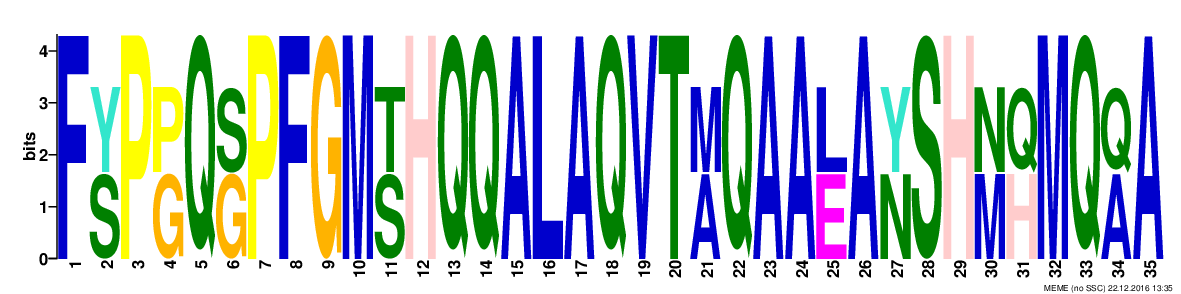 |
| **Motif 19** | 7.3e-004 | 7 | 18 | 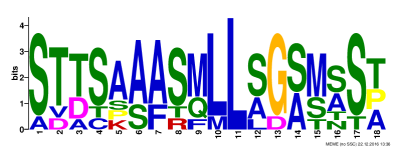 |
| **Motif 20** | 1.1e-003 | 7 | 15 | 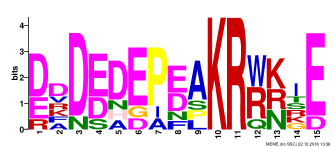 |
| **Motif 21** | 2.2e-003 | 2 | 41 | 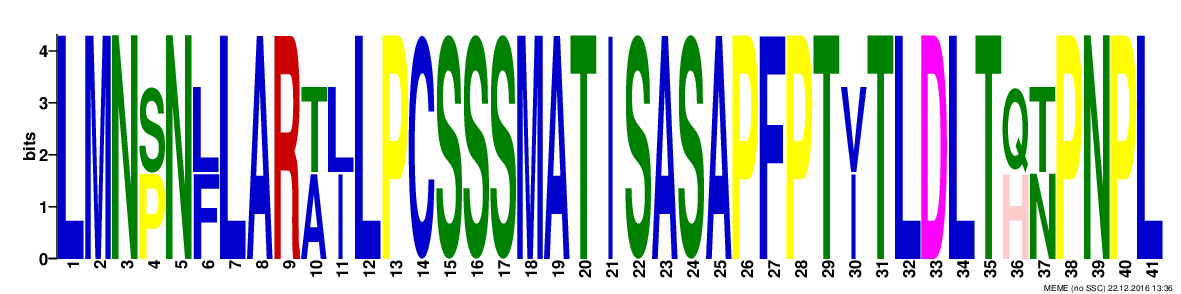 |
| **Motif 22** | 3.2e-003 | 5 | 8 | 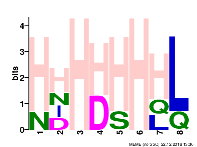 |
| **Motif 23** | 1.8e-004 | 10 | 8 | 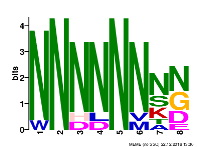 |
| **Motif 24** | 8.6e-003 | 4 | 15 | 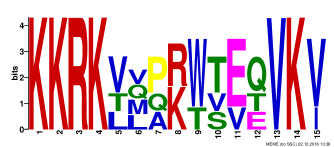 |
