# Supplementary material for: Identification and expression analyses of WRKY genes reveal their involvement in growth and abiotic stress response in watermelon (Citrullus lanatus)
Source: PLoS One. 2018 Jan 16;13(1):e0191308. doi: 10.1371/journal.pone.0191308 (PMC5770075; doi:10.1371/journal.pone.0191308)
Supplement: S6 Table — (DOCX) [file pone.0191308.s006.docx]

**Table S6 Number of WRKY TF gene family members present in major crop plants and horticultural plants.**

| Species | Genome size (Mb) | I | II | II-a | II-b | II-c | II-d | II-e | III | NG | Total |
| --- | --- | --- | --- | --- | --- | --- | --- | --- | --- | --- | --- |
| *Arabidopsis thaliana* [2] | 125 | 13 | 45 | 3 | 8 | 19 | 7 | 8 | 14 |  | 72 |
| *Oryza sativa* [21] | 466 | 13 | 42 | 4 | 8 | 14 | 6 | 10 | 47 |  | 102 |
| *Zea mays* [1] | 2300 | 10 | 78 | 7 | 11 | 29 | 14 | 17 | 31 |  | 119 |
| *Vitis vinifera* [44] | 490 | 12 | 39 | 3 | 8 | 15 | 7 | 6 | 6 | 2 | 59 |
| *Raphanus sativus* [18] | 402 | 31 | 74 | 6 | 15 | 27 | 14 | 12 | 20 | 1 | 126 |
| *Capsicum annuum* [12] | 3480 | 13 | 33 | 4 | 6 | 12 | 5 | 6 | 9 | 6 | 61 |
| *Cucumis sativus* [47] | 350 | 10 | 37 | 4 | 4 | 16 | 6 | 7 | 6 | 2 | 55 |
| *Citrullus lanatus* | 425 | 11 | 39 | 3 | 5 | 18 | 7 | 6 | 7 | 6 | 63 |
