# Supplementary figures and images for: Identification and expression analyses of WRKY genes reveal their involvement in growth and abiotic stress response in watermelon (Citrullus lanatus)
Source: PLoS One. 2018 Jan 16;13(1):e0191308. doi: 10.1371/journal.pone.0191308 (PMC5770075; doi:10.1371/journal.pone.0191308)

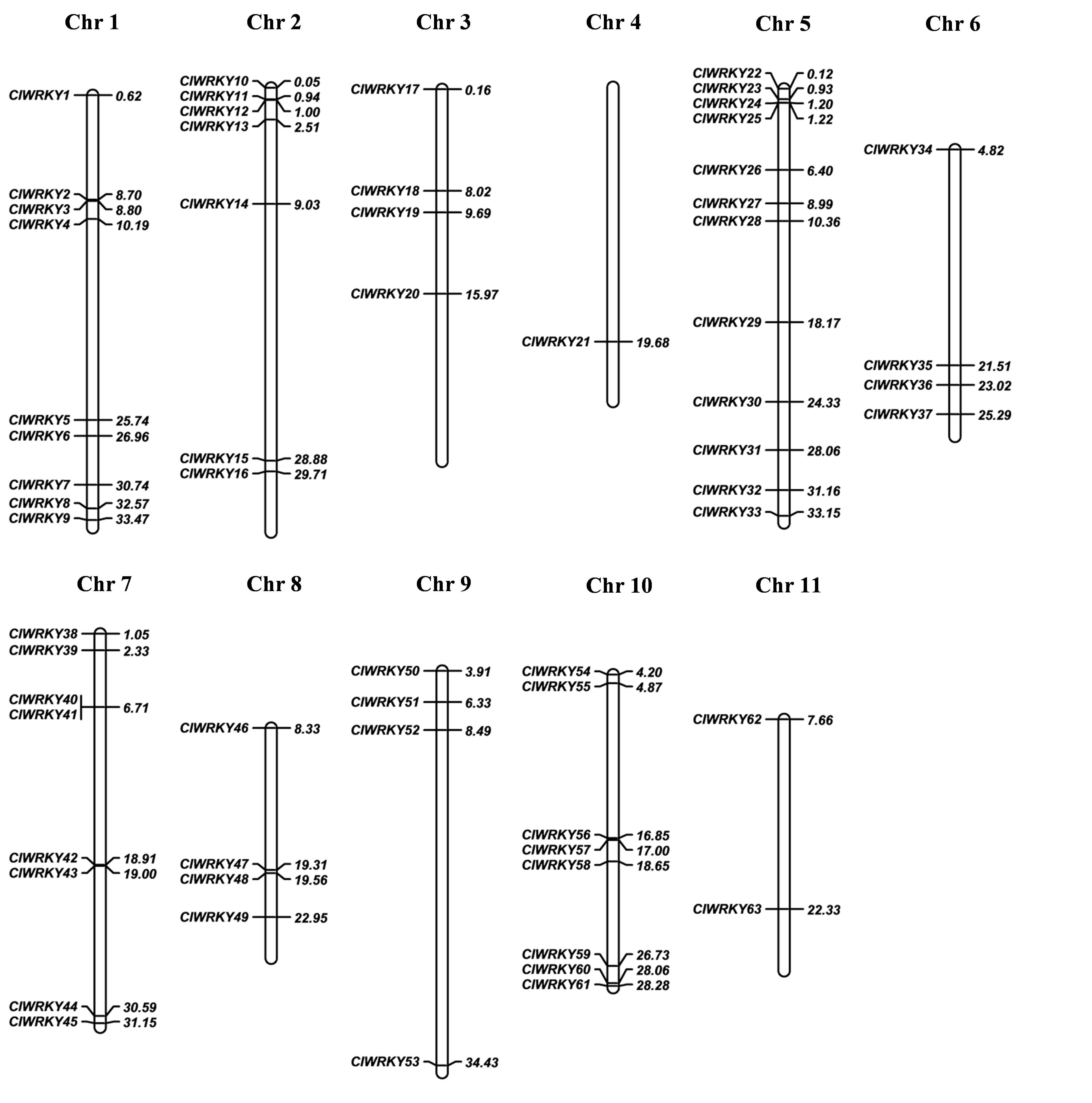

Supplement: S1 Fig — The numbers indicate the start site of WRKY genes located on chromosomes. (TIF) [file pone.0191308.s008.tif]
